# Data on the conservation potential of fish and coral populations in aquariums

**DOI:** 10.1016/j.dib.2018.12.083

**Published:** 2018-12-29

**Authors:** Rita da Silva, Dalia A. Conde

**Affiliations:** a Species360 Conservation Science Alliance, 7900 International Drive, Suite 1040, Bloomington, MN 55425, USA; b Interdisciplinary Centre on Population Dynamics, University of Southern Denmark, Denmark; c Department of Biology, University of Southern Denmark, Campusvej 55, 5230 Odense M, Denmark

## Abstract

The data presented in this article are related to the research article entitled “Assessing the Conservation Potential of Fish and Corals in Aquariums Globally”. This article unveils the number of fish and coral species held by members of the Species360 network, from records in the Zoological Information Management Systems (ZIMS). Furthermore, we provide data for species in ZIMS that are under the following global prioritization and conservation assessments: i) the Convention on International Trade in Endangered Species of Wild Fauna and Flora (CITES), ii) the International Union for Conservation of Nature (IUCN) Red List of Threatened Species, iii) Vulnerability to Climate Change, iv) Evolutionary Distinct and Globally Endangered (EDGE) and v) the Alliance for Zero Extinction (AZE). The material in this *Data in Brief* comprises the curated data and species overlaps in the mentioned prioritization schemes. It aims to facilitate further critical and extended analysis and to highlight the importance of record keeping and management of aquatic species in zoological institutions across the globe for the conservation of these species.

**Specifications table**

**Table:** 

Subject area	*Biology, Conservation*
More specific subject area	*Aquatic conservation, Species management, Captive husbandry, CITES, IUCN Red List of Threatened Species, Vulnerability to Climate Change, EDGE, AZE*
Type of data	*Table (word and excel documents)*
How data was acquired	*Document analysis, Online based databases*
Data format	*Filtered, analyzed*
Experimental factors	*Taxonomic standardization using R package ‘taxize’*
Experimental features	*Data curation and standardization among five global species assessments and prioritization schemes.*
Data source location	*Globally*
Data accessibility	*Data are with this article and at**https://www.species360.org/serving-conservation/aq-saving-species/*
Related research article	*‘Assessing the Conservation Potential of Fish and Corals in Aquariums Globally’*[1]

**Value of the data**

• This is the most comprehensive overlap of 3511 species of fish and corals with five global species conservation assessments and prioritization schemes. We aim to inform how many of the species in aquariums members of the Species360 global network registered in ZIMS align with those schemes.
• These data will assist further research on *ex situ* populations for species of conservation concern.
• These data could support the decision-making process of aquariums and zoological institutions on captive management and collection planning.
• The dataset may also assist in further assessments by identifying existing gaps and opportunities for conservation.

## 1 Data

The data incorporates 3511 species in aquariums with five conservation assessments and prioritization schemes.

## 2 Experimental design, materials, and methods

The creation of this dataset is based on an extensive analysis of six online datasets: i) species holdings from the Species360׳s Zoological Information Management System (ZIMS) [2], a centralized real-time system that manages data of more than 21,000 species held in zoos and aquariums; ii) The Convention on International Trade in Endangered Species of Wild Fauna and Flora (CITES) [3] an agreement between 183 countries to regulate international trade in plants and animals and their products; iii) the International Union for Conservation of Nature (IUCN) Red List [4], an assessment of species’ global risk of extinction and its threat status; iv) species Vulnerability to Climate Change [5], an assessment of species’ sensitivity, exposure and adaptive capacity to climate change; v) Evolutionary Distinct and Globally Endangered (EDGE) [6] that attributes scores according to the species’ evolutionary history and threat level and vi) species in the Alliance for Zero Extinction (AZE) [7], that prioritizes those species that are Critically Endangered or Endangered according to the IUCN Red List, with populations restricted to single sites.

The species names were standardized according to the accepted scientific name in Catalogue of Life (CoL) [8]. Species names and IUCN Red List status were automatically retrieved using the *R* package [9] *taxize* [10]. When accepted names across databases were not automatically standardized, we manually checked them. Based on the standardized species names, we cross-referenced databases using the *merge* function from *R*. We identified the species currently managed in a studbook in the European Association of Zoos and Aquariums (EAZA) [11] or in the American Association of Zoos and Aquariums (AZA) [12].

In Table 1 we show the number of species in each taxonomic class currently held in aquariums members of the Species360 network divided in IUCN Red List categories. In the same table we give the percentages of the number of species in aquariums from the total assessed by the IUCN Red List. The analysis of the number of species described and in aquariums with an evolutionary distinct (ED) score is given in Table 2, divided in arbitrary score categories. Species listed in more than one prioritization schemes are shown in Table 3, with species-specific indication of the IUCN Red List status, vulnerability to climate change, ED score and CITES index. In Table 4 we compiled the list of all species with active studbooks in EAZA and AZA together with their population sizes in aquariums members of the Species360 network and their current CITES listing. Table 5 has information on all the species currently in Species360 aquariums and information on all the studied prioritization schemes and population sizes. Table 4, Table 5 have two sheets, one with the respective data and other with the definition of the data on each column.

**Table 1:** Number of species in aquarium members of the Species360 global network and assessed by the IUCN Red List. Percentages are the number of species in aquariums relative to the total assessed by IUCN (%).

		EX	EW	CR	EN	VU	DD	NT	LC	NA
Actinopterygii	Aquariums	1	4	54	51	126	91	55	1373	1223
	IUCN	59	6	365	520	943	2193	366	7927	–
	%	2	67	15	10	13	4	15	17	–
Elasmobranchii	Aquariums	0	0	2	5	18	18	29	31	23
	IUCN	0	0	20	36	97	416	107	260	–
	%	–	–	10	14	19	4	27	12	–
Holocephali	Aquariums	0	0	0	0	0	0	0	1	0
	IUCN	0	0	0	0	0	20	3	23	–
	%	–	–	–	–	–	0	0	4	–
Myxini	Aquariums	0	0	0	0	0	1	0	2	0
	IUCN	0	0	1	1	3	21	2	31	–
	%	–	–	0	0	0	5	0	7	–
Sarcopterygii	Aquariums	0	0	0	0	0	0	0	2	3
	IUCN	0	0	1	0	1	0	0	3	–
	%	–	–	0	–	0	–	–	67	–
Anthozoa	Aquariums	0	0	2	3	28	1	40	77	106
	IUCN	0	0	7	28	199	292	174	292	–
	%	–	–	29	11	14	0.3	23	26	–

**Table 2:** Number of Evolutionary Distinct species of Anthozoa. In parenthesis are the percentage of the total described Anthozoa species. Evolutionary distinctiveness (ED) scores range from 5.18 to 75.76 for this class.

	ED < 8	8 < ED > 12	ED > 12	Total
Total described (CoL)	67	25	19	111
In Species360 aquariums	8	4	7	19

CoL: Catalogue of Life [8].

**Table 3:** List of species in aquariums listed in more than one of the prioritization schemes analysed: IUCN Red List threat categories, vulnerability to climate change assessment (i.e. high, low or unknown), Evolutionary Distinct score and CITES appendix by species.

**Species**	IUCN Red List Status	Vulnerability to climate change	ED Score	CITES
*Acanthastrea bowerbanki*	VU	Low	5.675922217	II
*Acropora loisetteae*	VU	Low	5.602749413	II
*Catalaphyllia jardinei*	VU	Low	16.16590613	II
*Cyphastrea ocellina*	VU	Low	7.612711002	II
*Euphyllia ancora*	VU	Low	9.461487289	II
*Euphyllia cristata*	VU	Low	7.013165598	II
*Euphyllia paradivisa*	VU	Low	7.011676007	II
*Galaxea astreata*	VU	Low	5.862920476	II
*Heliofungia actiniformis*	VU	Low	10.29736846	II
*Horastrea indica*	VU	Low	16.03679328	II
*Montastraea annularis*	EN	Unknown	8.67316998	II
*Pachyseris rugosa*	VU	Low	12.01815973	II
*Physogyra lichtensteini*	VU	Low	12.32702392	II
*Pocillopora verrucosa*	LC	Low	7.356618253	II
*Porites nigrescens*	VU	Low	5.512830088	II
*Turbinaria mesenterina*	VU	Low	16.5761365	II
*Turbinaria peltata*	VU	Low	16.42166023	II
*Turbinaria reniformis*	VU	Low	16.14904673	II

**Table 4:** List of species with active studbooks in EAZA (European Association of Zoos and Aquariums) and AZA (American Association of Zoos and Aquariums), the species IUCN Red List status, the studbook region, the population size in aquariums of the Species360 network and current CITES listing.

Species	Accepted scientific name according to Catalogue of Life (http://www.catalogueoflife.org/)
Studbook Names	Species names according to the institution responsible for the studbook (very detailed ex situ management records)
Class	Taxonomic class according to Catalogue of Life (http://www.catalogueoflife.org/)
Order	Taxonomic order according to Catalogue of Life (http://www.catalogueoflife.org/)
iucnStatus	Threatened category according to the IUCN Red List assessment (http://www.iucnredlist.org/)
Region	Zoological institution where the species is managed through a studbook
PopSize	Population size in institutions members of the Species360 network (https://www.species360.org/)
CITES.Listing.2017	CITES (Convention on International Trade in Endangered Species of Wild Fauna and Flora) listing appendix (https://www.cites.org/eng) downloaded in September 2017

**Table 5:** Complete list of species records and population sizes in institutions members of the Species360 network including species-specific information on the different prioritization schemes.

Species	Accepted scientific name according to Catalogue of Life (http://www.catalogueoflife.org/)
CITESAppendix.2017	Current CITES (Convention on International Trade in Endangered Species of Wild Fauna and Flora) listing appendix (https://www.cites.org/eng)
AZE	Indication of presence (1) or absence (0) in the listing of Alliance for Zero extinction (AZE) prioritization scheme (http://zeroextinction.org/)
CC	Indication of presence (1) or absence (0) in the assessment of species Vulnerability to Climate Change by Foden et al., 2013. Identifying the world׳s most climate change vulnerable species: a systematic trait-based assessment of all birds, amphibians and corals. PloS one 8, e65427.
EDGE	Indication of presence (1) or absence (0) in the listing of the Evolutionary Distinct and globally endangered prioritization scheme (https://www.edgeofexistence.org/)
IUCN	Threatened category according to the IUCN Red List assessment (http://www.iucnredlist.org/)
Species360	Indication of presence (1) or absence (0) in member institutions of Species360 (Population size in institutions members of the Species360 network (https://www.species360.org/)
PopSize.Sp360.2016	Population size in institutions members of the Species360 network (https://www.species360.org/) reported in ZIMS (data downloaded XXXX Date)
